# What Causes Desmoplastic Reaction in Small Intestinal Neuroendocrine Neoplasms?

**DOI:** 10.1007/s11912-022-01211-5

**Published:** 2022-05-13

**Authors:** Gowri M. Ratnayake, Faidon-Marios Laskaratos, Dalvinder Mandair, Martyn E. Caplin, Krista Rombouts, Christos Toumpanakis

**Affiliations:** 1 Neuroendocrine Tumour Unit - ENETS Centre of Excellence, Royal Free Hospital, London, NW3 2QG UK; 2 St Mark’s Academic Institute, Northwick Park, Watford Road, Harrow, HA1 3UJ Middlesex UK; 3 Regenerative Medicine & Fibrosis Group, Institute for Liver and Digestive Health, University College London, Royal Free Campus, London, NW3 2PF UK

**Keywords:** Desmoplasia, Fibrosis, Small intestinal neuroendocrine tumours, Tumour microenvironment, Growth factors

## Abstract

**Purpose of Review:**

Mesenteric desmoplasia in small intestinal neuroendocrine neoplasms (SINENs) is associated with increased morbidity and mortality. In this paper, we discuss the development of desmoplasia in SINENs.

**Recent Findings:**

The fibrotic reactions associated with these tumours could be limited to the loco-regional environment of the tumour and/or at distant sites. Mesenteric fibrotic mass forms around a local lymph node. Formation of desmoplasia is mediated by interactions between the neoplastic cells and its microenvironment via number of profibrotic mediators and signalling pathways. Profibrotic molecules that are mainly involved in the desmoplastic reaction include serotonin, TGFβ (transforming growth factor β) and CTGF (connective tissue growth factor), although there is some evidence to suggest that there are a number of other molecules involved in this process.

**Summary:**

Desmoplasia is a result of autocrine and paracrine effects of multiple molecules and signalling pathways. However, more research is needed to understand these mechanisms and to develop targeted therapy to minimise desmoplasia.

## Introduction

Desmoplasia could be defined as formation of fibrous connective tissue and forms a key component of solid tumour stroma [1, 2]. NENs are a heterogeneous group of tumours arising from the diffuse endocrine system and have a diverse range of biochemical, immunological properties and anatomical locations [3, 4].

A study involving 824 patients with SINENs showed that 88% of all participants had mesenteric lymph node metastases. Among 538 patients with stage IV SINENs in this study had fibrosis associated with mesenteric nodal metastases which were detected among 50% of the participants [5].

Small intestinal NENs (SINENs) are derived from the enterochromaffin cells and may give rise to carcinoid syndrome [6, 7] and result in loco-regional fibrosis with mesenteric desmoplasia and/or distant fibrotic processes such as carcinoid heart disease and retroperitoneal fibrosis [6–8].

Mesenteric desmoplasia is known to cause intestinal obstruction due to fibrotic adhesions or from mass effect. In addition, superior mesenteric vessels could be effected with arterial involvement causing small bowel ischaemia and superior mesenteric venous occlusion leading to ascites, small bowel venous ischaemia and varices [9, 10]. It is also reported that desmoplasia is associated with significantly increased morbidity and mortality [7, 9, 10].

Desmoplasia is radiologically diagnosed through its characteristic spoke wheel appearance with linear soft tissue opacities radiating outward from a central mesenteric mass [3, 9]. The severity of SINEN-induced mesenteric desmoplasia can be assessed radiologically by evaluating the number and thickness of strands radiating from the mesenteric mass [10].

This paper elaborates the current evidence on pathogenesis of desmoplasia in SINENs with an emphasis on tumour microenvironment, molecular mediators and downstream intracellular signalling pathways.

## Pathogenesis

It is believed that the nidus of mesenteric desmoplasia is a small bowel lymph node. Desmoplasia is the result of complex interactions mediated via profibrotic and anti-fibrotic factors between the SINEN cells and the tumour microenvironment [3]. These molecular mediators are likely to be produced in the metastatic tumour cells in the mesentery and exert their actions via autocrine and/or paracrine fashion to mediate a local fibrotic reaction [3].

NENs do not only comprise proliferating cancer cells, but also consist of a plethora of heterogeneous non-cancerous cells and a non-cellular matrix which composite the tumour microenvironment [2, 11•, 12–14]. There is also evidence to suggest that the cross-talk between these two compartments is essential for tumour proliferation, modulation of the tumour behaviour and treatment responsiveness. In addition, it is known that the tumour microenvironment and cellular components have similarities to the wound healing process [15].

## Tumour Microenvironment

The tumour microenvironment consists of stromal cells, extracellular matrix (ECM), endothelial cells and inflammatory cells [2, 11•, 12–14].

The stromal cells in the tumour microenvironment predominately consist of fibroblasts. However, these fibroblasts are of a modified activated phenotype and are best named as cancer-associated fibroblasts (CAF) [11•, 16••, 17], and the activated form of CAFs could be identified by expression of α smooth muscle actin (α-SMA) and proliferation [14].

SINENs demonstrate a high rate of α smooth muscle actin expressing fibroblasts than the other NENs [16••, 17]. A study looking in to markers of fibroblast activation in the fibrotic stroma of the SINENs demonstrated increased presence of α smooth muscle actin producing fibroblasts [18] providing evidence that the increased number of activated fibroblasts plays an important role in SINEN-induced desmoplastic process. The transformation of epithelial derived fibroblasts to α smooth muscle actin expressing myofibroblasts/CAF is a distinct feature of cancer-associated fibrosis [19].

The ECM consists of a complex network of crosslinked collagens, glycoproteins and water that continuously undergoes remodelling through enzymatic and non-enzymatic post-translational changes [13, 18]. The overexpression of fibroblasts in the stroma, and a disbalance between MMPs and TIMPs, leads to increased deposition of collagen, thus promoting desmoplasia.

The inflammatory cells in the tumour microenvironment is known to contain a large number of tumour-associated macrophages which effectively supress the immune system and promote tumour growth and fibrosis by secreting profibrotic factors [16••, 20]. In SINENs, the number of leucocyte infiltration in the tumour microenvironment is less in comparison to the other solid organ tumours, even though the existing macrophages stained positively for TGFβ and platelet-derived growth factor (PDGF) [3, 11•, 14, 16••, 21].

## Fibrotic Growth Factors

SINENs originate from the enterochrromafin cells of the diffuse endocrine system and secrete various amines and peptides promoting the tumour growth via autocrine and paracrine effects [4, 7]. Some of these molecules also have pro-fibrotic activity and play an important role in desmoplasia via enabling the cross-talk between the tumour cells and the tumour microenvironment [3, 16••]. Most of the studies describing the role of these factors in SINENs show the presence of these fibrotic growth factors on the tumour cells and/or in the stromal fibroblasts and the presence of their receptors on the fibroblast. However, there is some evidence through in vitro co-culture methods that these molecules are responsible for the cross-talk between the tumour cells and other cellular components in the tumour microenvironment and these interactions are important for tumour growth, fibrosis and angiogenesis [22].

Serotonin (5 HT) is an amine derived from the amino acid tryptophan and is produced in SINENs and excreted by the body predominantly in the urine in the form of 5 hydroxyindole acetic acid (5 HIAA) [3, 6]. Serotonin acts via 5-hydroxytryptamine (5 HT) receptors of which 7 receptor subtypes have been identified. Although 5 HT-3 receptor is a ligand-gated ion channel, the rest of the 5 HT receptors are G protein–coupled receptors [3]. It is recognised that patients with an elevated level of urinary 5 HIAA or carcinoid syndrome are known to have a higher risk of developing desmoplasia [23] implicating a possible correlation between the serotonin and desmoplasia. Furthermore, 5HT receptor agonist therapy is known to cause retroperitoneal, myocardial, aortic and pleuro-pulmonary fibrosis demonstrating the role of serotonin in the role of fibrosis [3, 16••, 24, 25].

Svejda and colleagues developed an in vitro co-culture model containing small intestinal neuroendocrine tumour cells (KRJ-I) [22], which were recently described as to have lymphoblastoid immunophenotype [26] in one compartment and fibroblast cells (HEK 293) in another compartment and demonstrated that there are molecules involved in the cross-talk between these 2 cellular compartments [22]. This model revealed that serotonin plays an important role in the cross-talk between tumour cells and fibroblasts. Furthermore, there is also evidence that serotonin inhibits fibrinolysis further promoting fibrogenesis [3, 27]. Serotonin secreted by SINENs may have autocrine and paracrine effects on tumour cells and fibroblasts residing in the tumour microenvironment respectively leading to tumour growth and fibrosis.

Transforming growth factor-β (TGF-β) is also a pro-fibrotic agent that is secreted by the SINENs [3, 16••, 27]. All 3 forms of TGF-β (β1, β2, β3) production have been demonstrated in SINEN cells along with TGF-β1 and TGF-β2 receptors. Furthermore, TGF-β2 receptors are located on the stromal fibroblasts allowing the tumour cross-talk to occur efficiently [3, 16••, 22]. Paracrine effects of TGF-β secreted by tumour cells on stromal fibroblasts stimulate their differentiation and subsequent desmoplasia. In addition, TGF-β produced by fibroblasts may act in an autocrine manner [16••, 27].

Connective tissue growth factor (CTGF) is a downstream mediator of TGF-β and serotonin [14] and it is expressed by > 50% of SINEN cells [15, 16••]. While CTGF acts by binding to CTGF receptor, it could also potentiate its effects by binding to insulin-like growth factor (IGF) receptors. Furthermore, activation of the CTGF pathway augments the activation of IGF and endothelial growth factor (EGF) receptor signalling cascades promoting further collagen production and fibroblast proliferation [28].

CTGF may enhance profibrotic actions of TGF-β, FGF and EGF by stimulating fibroblast proliferation, collagen production and differentiation of fibroblasts into α smooth muscle actin producing fibroblasts. The location of these CTGF-secreting neoplastic cells was identified close to the α smooth muscle actin producing fibroblasts which could also suggest a potential role of CTGF to desmoplastic reaction of SINENs [3, 16••, 17, 18]. Moreover, circulating CTGF levels were found to be significantly higher in patients with SINEN in comparison to patients with gastric NENs and healthy controls [17].

Platelet-derived growth factor (PDGF) is a strong profibrotic agent and exerts its effect via PDGF-α and PDGF-β receptors and also stimulates the proliferation of fibroblasts. In SINENs, PDGFβ receptors are found in 65–85% of the cases and the receptors are probably on α smooth muscle actin producing fibroblasts but not on tumour cells. Evidence suggests that the tumour cells and the macrophages in the tumour microenvironment induce PDGFRβ expression on adjacent fibroblasts and upregulate PDGFRβ activity in a paracrine fashion promoting PDGF-mediated fibrogenesis [29].

Transforming growth factor α (TGF α) is a peptide related to epithelial growth factor (EGF) and is known to express its effects via the EGF receptors. While it is overly expressed by the SINEN cells (> 90%), it also plays a crucial role in fibrosis and induce tumoural angiogenesis and proliferation. TGF-α produced by tumour cells acts on fibroblasts via the EGF receptors located on the fibroblasts [3, 16••, 30].

Fibroblast growth factor (FGF) type 2/acidic FGF is known to be expressed by the SINEN cells and the adjacent stromal cells indicating its possible role in fibrosis [30, 31]. However, more evidence is needed to determine whether it plays a significant process in desmoplastic reaction associated with SINENs.

Insulin-like growth factors (IGFs) 1 and 2 are known to increase the proliferation of fibroblasts in chick embryos, mammals and humans [32]. These growth factors exert their effects by binding to IGF 1 and 2 receptors and the concentration and the bioavailability of IGF is controlled through IGF-binding proteins (IGFBP) [33]. IGF1, IGF1 receptors and IGFBP are being expressed by most stromal cells [34, 35]. However, expression of the IGF2 and IGF2 receptors on stromal cells is rare [36]. Further evidence is needed to elicit their role in the pathogenesis of desmoplasia.

Substance P (SP) is tachykinin that is secreted by SINENs and is a known mediator of carcinoid syndrome [6]. In addition, SP is known to augment cytokine-induced fibroblast proliferation [37]. Natural killer (NK) cells are known to have an anti-fibrotic action in the intestines and in the liver by inhibiting TGF-β activity [16••, 36, 38]. SP is potent antagonist of NK1 receptor and thus potentiates fibrosis by increasing TGF-β activity although there is insufficient data to suggest whether this mechanism induces fibrosis in SINENs.

Nerve growth factor (NGF) is known to potentiate effects of TGF-β and is considered a profibrotic agent. SINENs are known to express NGF [39, 40]. In contrary to its profibrotic activity, its levels are known to be lower among SINENs with extensive mesenteric angiopathy and fibrosis in comparison to those with limited fibrosis [31, 41].

Histamine produced by the SINENs is considered as a mediator of carcinoid syndrome [6] and is also know to enhance the cytokine-induced fibroblast activation [37].

## Complex Intracellular Signalling Pathways

While the above details express the potential roles of individual profibrotic growth factors in the development of desmoplasia, there is developing evidence to suggest that complex cross-talk between the above molecules and post-receptor signalling intracellular cascades is involved in the development of SINEN-associated desmoplasia [3, 16••, 42]. Interestingly, a recent study from our centre revealed that the integrin signalling pathway is involved in the cross-talk of neuroendocrine tumour cells and stromal cells and may have a dual role in carcinogenesis and fibrogenesis [43]. This study involved in vitro co-culture experiments with neuroendocrine tumour KRJ-I cells and stromal HEK293 cells and investigated genes and pathways involved in their cross-talk. The integrin signalling pathway, which was activated in KRJ-I cells, was further confirmed in human tissue at RNA and protein levels and several genes involved in this pathway were upregulated within the fibrotic mesenteric mass. This pathway is interesting because of its roles in carcinogenesis and fibrogenesis in various tumours [43]. Similar to other cancers, our current understanding is that there may therefore be a bidirectional effect whereby the interaction of cancer cells and fibroblasts promotes not only desmoplasia, but also may enhance the growth of cancer cells and their metastatic potential. This is confirmed by our clinical observational studies on SINENs in which patients with mesenteric desmoplasia had a shorter overall survival which was independent of other factors and not associated with abdominal complications, but with disease progression [10].

Moreover, cancer-associated fibrosis is known to be mediated via complex intracellular signalling pathways namely Hedgehog (Hh), Notch and Wnt/β-catenin and it is worth to discuss the evidence for these complex pathways in the context of SINEN-associated desmoplasia [16••, 44].

Hh cascade is activated by several ligands (Sonic, Desert and Indian) which bind to Patch receptors stimulating production of transcription factors (Gli 1–3). Gli 1 transcriptional factor increases the expression of Snail which in turn leads to suppression of E-cadherin promoting epithelial to mesenchymal transition (EMT) and formation of myofibroblasts and subsequent desmoplasia [15, 16••]. Fendrich and colleagues examined 39 specimens of SINENs using immunohistochemical staining and revealed that Snail expression was detected in 59% of cases, intratumour Sonic Hh was expressed in 73% and combined Snail and Sonic Hh was expressed among 53%. As expected, immunofluorescence staining revealed downregulation of E-cadherin on Snail-positive cells, leading to the possible hypothesis that the Hh pathway may induce fibrosis [45•]. This data provides some insight to Hh pathway activation in the development of SINEN-associated desmoplasia. However, there is also evidence to suggest that TGF-β is known to increase the Gli 1 expression, which may be independent of Hh pathway [15].

Notch signalling also drives the epithelial to mesenchymal transition increasing the production of fibroblasts/myofibroblast population and thus promoting extensive fibrosis. In addition, activation of this pathway also stimulated TGFβ production which may further stimulate the fibrotic process [15]. A study demonstrated that by stimulating Notch 1 production on pancreatic NEN cells (BON cells) lead to reduced production of serotonin by these cells through a multi-step signal transmission cascade [46]. Another study on Notch pathway activation in gastrointestinal NENs suggested that there is 89% reduction of serotonin synthesis with equivalent reduction of serotonin reactive cells and repression of tryptophan hydroxylase, i.e. the enzyme catalysing the rate limiting step of serotine biosynthesis, mRNA synthesis [47]. As such, Notch pathway activation at least theoretically may exert some anti-fibrotic effect by reducing serotonin production. However, later on, a study involving 31 SINENs (ileal) demonstrated that none of these tumours expresses Notch discrediting the role of Notch signalling pathway in the development of fibrosis in SINENs [48].

β catenin levels in the cytoplasm are maintained by binding to a destructive protein complex. Upon activation of Wnt signaling pathway, β catenin levels in the cytoplasm rise and then these molecules translocate to the nucleus activating target gene transcription [15]. However, only membranous rather than intracytoplasmic β catenin expression had been demonstrated in SINENs [16••, 45•]. Moreover, the severity of desmoplasia was not associated with the degree of β catenin expression in SINENs. Hence, the current evidence suggests only a minor role of Wnt/β catenin signalling pathway in the development of desmoplasia [49].

Despite our existing knowledge of the pathogenesis of mesenteric fibrosis, there are hardly any medical therapeutic strategies to treat mesenteric desmoplasia to the date [15]. Available medical therapy for management of NENs and carcinoid syndrome includes somatostatin receptor analogues, serotonin synthesis inhibitors (telotristat), 5 HT receptor antagonists (cyproheptidine) and tyrosine kinase inhibitors [6] which have not been specifically assessed for their potential anti fibrotic effect [16••].

Therefore, the treatment of mesenteric fibrosis mainly involves surgical resection of the mesenteric fibrotic mass, palliative bypass surgeries in the presence of inoperable tumour and obstruction and stenting [8].

## Conclusions

SINEN-associated desmoplasia is associated with increased morbidity and mortality. Desmoplasia is the result of a complex interaction between SINEN cells and the tumour microenvironment mediated via a plethora of molecules potentiating their profibrotic effects independently or via a cascade of downstream signalling pathways. The key molecules known to be involved in the pathogenesis of SINEN-associated desmoplasia are serotonin, TGFβ and CTGF. Yet, to date, there is no effective medical therapy to treat or reverse this phenomenon. Further research into the pathophysiology of this desmoplastic reaction should provide insights into future development of targeted anti fibrotic therapies for SINEN-associated desmoplasia.

## References

[1] Kate SA, Hall BM. Desmoplasia. In Encyclopedia of Cancer (pp. 1093–1095). Springer Berlin Heidelberg. 2011. 10.1007/978-3-642-16483-5_1580.

[2] Chandler C, Liu T, Buckanovich R, Coffman LG. The double edge sword of fibrosis in cancer. Transl Res. Mosby Inc. 2019. 10.1016/j.trsl.2019.02.006. 10.1016/j.trsl.2019.02.006 PMC6545239 30871956

[3] Laskaratos FM, Rombouts K, Caplin M, Toumpanakis C, Thirlwell C, Manir D. Neuroendocrine tumors and fibrosis: an unsolved mystery? Cancer. John Wiley and Sons Inc. 2017. 10.1002/cncr.31079. 10.1002/cncr.31079 29112233

[4] Caplin ME, Ratnayake GM. Diagnostic and therapeutic advances in neuroendocrine tumours. Nat Rev Endocrinol. Nature Research. 2021. 10.1038/s41574-020-00458-x. 10.1038/s41574-020-00458-x 33335329

[5] Daskalakis K, Karakatsanis A, Stålberg P, Norlén O, Hellman P (2017). Clinical signs of fibrosis in small intestinal neuroendocrine tumours. Br J Surg.

[6] Ratnayake G, Toumpanakis C. Carcinoid syndrome and its sequelae. Current Opinion in Endocrine and Metabolic Research. Elsevier Ltd. 2021. 10.1016/j.coemr.2021.02.006.

[7] Modlin IM, Shapiro MD, Kidd M (2004). Carcinoid tumors and fibrosis: an association with no explanation. Am J Gastroenterol.

[8] Druce M, Rockall A, Grossman AB (2009). Fibrosis and carcinoid syndrome: from causation to future therapy. Nat Rev Endocrinol.

[9] Laskaratos FM, Mandair D, Hall A, Alexander S, von Stempel C, Bretherton J, … Toumpanakis C. Clinicopathological correlations of mesenteric fibrosis and evaluation of a novel biomarker for fibrosis detection in small bowel neuroendocrine neoplasms. Endocrine. 2020; 67(3), 718–726. 10.1007/s12020-019-02107-4. 10.1007/s12020-019-02107-4 PMC7054371 31598848

[10] Laskaratos FM, Diamantopoulos L, Walker M, Walton H, Khalifa M, El-Khouly F, … Mandair D. Prognostic factors for survival among patients with small bowel neuroendocrine tumours associated with mesenteric desmoplasia. Neuroendocrinology. 2018; 106(4), 366–380. 10.1159/000486097. 10.1159/000486097 29320779

[11] • Cives M, Pelle E, Quaresmini D, Rizzo FM, Tucci M, Silvestris F. The tumor microenvironment in neuroendocrine tumors: biology and therapeutic implications. Neuroendocrinology. S. Karger AG. 2019. 10.1159/000497355. **This publication reports the importance of tumour microenviorenemnt on the tumour growth, angiogenesis and fibrosis and the factors influencing there processes**. 10.1159/000497355 30699437

[12] Albrengues J, Bourget I, Pons C, Butet V, Hofman P, Tartare-Deckert S, … Gaggioli C. LIF mediates proinvasive activation of stromal fibroblasts in cancer. Cell Rep. 2014; 7(5), 1664–1678. 10.1016/j.celrep.2014.04.036. 10.1016/j.celrep.2014.04.036 24857661

[13] Hanahan D, Weinberg RA (2011). Hallmarks of cancer: the next generation. Cell.

[14] Piersma B, Hayward MK, Weaver VM. Fibrosis and cancer: a strained relationship. Biochimica et Biophysica Acta - Reviews on Cancer. Elsevier B.V. 2020. 10.1016/j.bbcan.2020.188356. 10.1016/j.bbcan.2020.188356 PMC7733542 32147542

[15] Rybinski B, Franco-Barraza J, Cukierman E. The wound healing, chronic fibrosis, and cancer progression triad. Physiol Genomics. American Physiological Society. 2014. 10.1152/physiolgenomics.00158.2013. 10.1152/physiolgenomics.00158.2013 PMC4035661 24520152

[16] •• Blažević A, Hofland J, Hofland LJ, Feelders RA, De Herder WW. Small intestinal neuroendocrine tumours and fibrosis: an entangled conundrum. Endocr-Relat Cancer. BioScientifica Ltd. 2018. 10.1530/ERC-17-0380. **Authors review the evidence on elements of tumour microenvironment and the growth factors in pathogenesis of the mesenteric fibrosis and the available/potential targeted anti fibrotic therapy**. 10.1530/ERC-17-0380 29233841

[17] Kidd M, Modlin IM, Shapiro MD, Camp RL, Mane SM, Usinger W, Murren JR (2007). CTGF, intestinal stellate cells and carcinoid fibrogenesis. World J Gastroenterol.

[18] Cunningham JL, Tsolakis AV, Jacobson A, Janson ET (2010). Connective tissue growth factor expression in endocrine tumors is associated with high stromal expression of α-smooth muscle actin. Eur J Endocrinol.

[19] Yamauchi M, Barker TH, Gibbons DL, Kurie JM. The fibrotic tumor stroma. J Clin Investig. American Society for Clinical Investigation. 2018. 10.1172/JCI93554. 10.1172/JCI93554 PMC5749516 29293090

[20] Wynn TA (2008). Cellular and molecular mechanisms of fibrosis. J Pathol.

[21] Papanicolaou V, Papanicolaou V, Oberg K, Heldin CH, Funa K (1992). Expression of platelet-derived growth factor and its receptors in neuroendocrine tumors of the digestive system. Can Res.

[22] Svejda B, Kidd M, Giovinazzo F, Eltawil K, Gustafsson BI, Pfragner R, Modlin IM (2010). The 5-HT2B receptor plays a key regulatory role in both neuroendocrine tumor cell proliferation and the modulation of the fibroblast component of the neoplastic microenvironment. Cancer.

[23] Rodríguez Laval V, Pavel M, Steffen IG, Baur AD, Dilz LM, Fischer C, … Denecke T. Mesenteric fibrosis in midgut neuroendocrine tumors: functionality and radiological features. Neuroendocrinology. 2018; 106(2), 139–147. 10.1159/000474941. 10.1159/000474941 28384635

[24] Reimund E (1987). Methysergide and retroperitoneal fibrosis. Lancet.

[25] Rothman RB, Baumann MH, Savage JE, Rauser L, McBride A, Hufeisen SJ, Roth BL (2000). Evidence for possible involvement of 5-HT(2B) receptors in the cardiac valvulopathy associated with fenfluramine and other serotonergic medications. Circulation.

[26] Hofving T, Arvidsson Y, Almobarak B, Inge L, Pfragner R, Persson M, Stenman G, Kristiansson E, Johanson V, Nilsson O (2018). The neuroendocrine phenotype, genomic profile and therapeutic sensitivity of GEPNET cell lines. Endocr Relat Cancer.

[27] Observations on pathogenesis of carcinoid heart disease and tanning of fluorescent fibrin by 5-hydroxytryptamine and ceruloplasmin. JAMA J Am Med Assoc. 1963; 183(4):181. 10.1001/jama.1963.03700040115074. 10.1093/ajcp/39.1.46 13966804

[28] Leask A, Abraham DJ (2006). All in the CCN family: essential matricellular signaling modulators emerge from the bunker. J Cell Sci.

[29] Witsch E, Sela M, Yarden Y. Roles for growth factors in cancer progression. Physiology. American Physiological Society. 2010;25:85–101. 10.1152/physiol.00045.2009. 10.1152/physiol.00045.2009 PMC3062054 20430953

[30] Wimmel A, Wiedenmann B, Rosewicz S (2003). Autocrine growth inhibition by transforming growth factor β-1 (TGFβ-1) in human neuroendocrine tumour cells. Gut.

[31] Chaudhry A, Funa K, Öberg K (1993). Expression of growth factor peptides and their receptors in neuroendocrine tumors of the digestive system. Acta Oncol.

[32] Zhang PJ, Furth EE, Cai X, Goldblum JR, Pasha TL, Min KW (2004). The role of β-catenin, TGFβ3, NGF2, FGF2, IGFR2, and BMP4 in the pathogenesis of mesenteric sclerosis and angiopathy in midgut carcinoids. Hum Pathol.

[33] La Rosa S, Chiaravalli AM, Capella C, Uccella S, Sessa F (1997). Immunohistochemical localization of acidic fibroblast growth factor in normal human enterochromaffin cells and related gastrointestinal tumours. Virchows Arch.

[34] Froesch ER, Schmid C, Schwander J, Zapf J (1985). Actions of insulin-like growth factors. Annu Rev Physiol.

[35] Poreba E, Durzynska J. Nuclear localization and actions of the insulin-like growth factor 1 (IGF-1) system components: transcriptional regulation and DNA damage response. Mutat Res-Rev Mutat Res. Elsevier B.V. 2020. 10.1016/j.mrrev.2020.108307. 10.1016/j.mrrev.2020.108307 32430099

[36] Nilsson O, Wängberg B, Mcrae A, Dahlström A, Ahlman H (1993). Growth factors and carcinoid tumours. Acta Oncol.

[37] Koon HW, Shih D, Karagiannides I, Zhao D, Fazelbhoy Z, Hing T, Xu H, Lu B, Gerard N, Pothoulakis C (2010). Substance P modulates colitis-associated fibrosis. Am J Pathol.

[38] Wulbrand U, Remmert G, Zöfel P, Wied M, Arnold R, Fehmann HC (2000). mRNA expression patterns of insulin-like growth factor system components in human neuroendocrine tumours. Eur J Clin Invest.

[39] Wan Y, Meng F, Wu N, Zhou T, Venter J, Francis H, … Alpini G. Substance P increases liver fibrosis by differential changes in senescence of cholangiocytes and hepatic stellate cells. Hepatology. 2017. 66(2), 528–541. 10.1002/hep.29138. 10.1002/hep.29138 PMC5519428 28256736

[40] Katayama I, Nishioka K (1997). Substance P augments fibrogenic cytokine-induced fibroblast proliferation: possible involvement of neuropeptide in tissue fibrosis. J Dermatol Sci.

[41] La Rosa S, Uccella S, Capella C, Chiaravalli AM, Sessa F (1998). Localization of acidic fibroblast growth factor, fibroblast growth factor receptor-4, transforming growth factor-α, and epidermal growth factor receptor in human endocrine cells of the gut and related tumors: An immunohistochemical study. Appl Immunohistochem.

[42] Boilly B, Faulkner S, Jobling P, Hondermarck H. Nerve dependence: from regeneration to cancer. Cancer Cell. Cell Press. 2017. 10.1016/j.ccell.2017.02.005. 10.1016/j.ccell.2017.02.005 28292437

[43] Vizza D, Perri A, Toteda G, Lupinacci S, Leone F, Gigliotti P, Lofaro D, La Russa A, Bonofiglio R (2015). Nerve growth factor exposure promotes tubular epithelial-mesenchymal transition via TGF-β1 signaling activation. Growth Factors (Chur, Switzerland).

[44] Aschner Y, Downey GP (2016). Transforming growth factor-B: master regulator of the respiratory system in health and disease. Am J Respir Cell Mol Biol Am Thorac Soc.

[45] • Laskaratos FM, Levi A, Schwach G, Pfragner R, Hall A, Xia D, … Rombouts K. Transcriptomic profiling of in vitro tumor-stromal cell paracrine crosstalk identifies involvement of the integrin signaling pathway in the pathogenesis of mesenteric fibrosis in human small intestinal neuroendocrine neoplasms. Front Oncol. 2021;11. 10.3389/fonc.2021.629665. **This research evaluated the signalling mechanisms involved in the cross-talk between the SINEN-derived cell cultures and the fibroblast-derived cell cultures including the changes on gene expression on each of these cell lines**. 10.3389/fonc.2021.629665 PMC7943728 33718208

[46] Hoeft K, Kramann R. Developmental signaling and organ fibrosis. Curr Pathobiol Rep. Springer. 2017. 10.1007/s40139-017-0136-8.

[47] Fendrich V, Waldmann J, Esni F, Ramaswamy A, Mullendore M, Buchholz M, … Feldmann G. Snail and Sonic Hedgehog activation in neuroendocrine tumors of the ileum. Endocr-Relat Cancer. 2007; 14(3), 865–874. 10.1677/ERC-07-0108. 10.1677/ERC-07-0108 17914115

[48] Kunnimalaiyaan M, Traeger K, Chen H. Conservation of the Notch1 signaling pathway in gastrointestinal carcinoid cells. Am J Physiol-Gastrointest Liver Physiol. 2005;289(4 52–4). 10.1152/ajpgi.00146.2005. 10.1152/ajpgi.00146.2005 16160079

[49] Nakakura EK, Sriuranpong VR, Kunnimalaiyaan M, Hsiao EC, Schuebel KE, Borges MW, … Ball DW. Regulation of neuroendocrine differentiation in gastrointestinal carcinoid tumor cells by notch signaling. J Clin Endocrinol Metab. 2005; 90(7), 4350–4356. 10.1210/jc.2005-0540. 10.1210/jc.2005-0540 15870121

